# NR4A1 Promotes PDGF-BB-Induced Cell Colony Formation in Soft Agar

**DOI:** 10.1371/journal.pone.0109047

**Published:** 2014-09-30

**Authors:** Glenda Eger, Natalia Papadopoulos, Johan Lennartsson, Carl-Henrik Heldin

**Affiliations:** Ludwig Institute for Cancer Research, Uppsala University, Uppsala, Sweden; University of Patras, Greece

## Abstract

The fibroblast mitogen platelet-derived growth factor -BB (PDGF-BB) induces a transient expression of the orphan nuclear receptor NR4A1 (also named Nur77, TR3 or NGFIB). The aim of the present study was to investigate the pathways through which NR4A1 is induced by PDGF-BB and its functional role. We demonstrate that in PDGF-BB stimulated NIH3T3 cells, the MEK1/2 inhibitor CI-1040 strongly represses NR4A1 expression, whereas Erk5 downregulation delays the expression, but does not block it. Moreover, we report that treatment with the NF-κB inhibitor BAY11-7082 suppresses NR4A1 mRNA and protein expression. The majority of NR4A1 in NIH3T3 was found to be localized in the cytoplasm and only a fraction was translocated to the nucleus after continued PDGF-BB treatment. Silencing NR4A1 slightly increased the proliferation rate of NIH3T3 cells; however, it did not affect the chemotactic or survival abilities conferred by PDGF-BB. Moreover, overexpression of NR4A1 promoted anchorage-independent growth of NIH3T3 cells and the glioblastoma cell lines U-105MG and U-251MG. Thus, whereas NR4A1, induced by PDGF-BB, suppresses cell growth on a solid surface, it increases anchorage-independent growth.

## Introduction

Platelet-derived growth factor (PDGF) is a key mitogen for cells of mesenchymal origin, with important functions during embryonic development and wound healing. The biologically active isoforms of PDGF are disulfide-bonded dimers of A, B, C or D polypeptide chains, i.e. PDGF-AA, -BB, -AB, -CC and -DD, which bind to structurally related α- and β-tyrosine kinase receptors (PDGFRα and PDGFRβ, respectively). The two PDGFRs have different ligand binding specificities; PDGFRα binds PDGF A-, B- and C- chains, whereas PDGFRβ binds B- and D-chains [1]. Binding of the dimeric PDGF isoforms results in homo- or hetero-dimerization of the receptors and subsequent autophosphorylation of tyrosine residues in their intracellular parts. The autophosphorylation activates the kinase activity of the receptors and the phosphorylated tyrosine residues serve as interaction sites for SH2-domain-containing signal transduction proteins, which relay or modulate several signaling pathways. Examples include GRB2/SOS1 which activates extracellular signal-regulated kinase 1 and 2 (Erk1/2) MAP kinase, phosphatidylinositol 3-kinase (PI3-kinase), phospholipase C-γ, STAT family members, members of the Src family of tyrosine kinases, and the protein tyrosine phosphatase SHP-2 [1] [2]. These signaling pathways promote cell proliferation, migration and survival. Overactivity of PDGF pathways is implicated in diseases involving excessive cell growth, including malignancies, cardiovascular disease and fibrosis [3].

The MAP-kinase pathways activated by PDGF include Erk1/2, Erk5, c-Jun N-terminal kinase (JNK), and p38 [4] [5]. Erk5, unlike the other MAP-kinases, has an extended, unique C-terminal region with a bipartite nuclear localization signal (NLS) [6], and a transcriptional activation domain [7], suggesting that Erk5 may function both as a kinase and as a transcription factor. Activated MAP-kinases phosphorylate several substrates, including cytosolic signaling proteins and transcription factors affecting cell proliferation, survival and migration.

Nuclear receptors function as ligand-activated transcription factors; however, there are several examples of so called orphan nuclear receptors for which no ligand has been identified. The function of orphan nuclear receptors can be regulated by expression levels and/or post-translational modifications, such as phosphorylation. NR4A1 (Nur77, TR3, NGF1IB) is an example of an orphan nuclear receptor that can be phosphorylated by Erk1/2, Erk5 and JNK MAP-kinases, as well as other kinases such as Akt, Rsk, GSK3β and DNA-PK [8]. NR4A1 belongs to a family which also encompasses NR4A2 (NURR1) and NR4A3 (NOR-1) characterized by a conserved DNA binding domain that suggests redundancy among them. Notably, members of the NR4A1 family is frequently found to be induced by growth factors [11] [9]. Both phosphorylation and acetylation have been shown to control NR4A1 stability and/or subcellular localization [10] [11] [12] [13]. Multiple and sometimes opposing functions of NR4A1 have described in different cell types which may be related to differences in subcellular localization. Overexpression of NR4A1 resulted in increased survival and proliferation of human umbilical vein endothelial cells [14]. On the other hand, an apoptotic effect was associated with a mitochondrial localization of NR4A1, where it converted BCL-2 from an anti- to a pro-apoptotic protein [15] [16] [17]. Moreover, it has been show that NR4A1 is involved in T cell receptors-mediated apoptosis in immature thymocytes [18] [19] and roles for NR4A1 has also been described in metabolism [20], steroidogenesis [21] [22], as well as in suppression of smooth muscle cells proliferation by upregulating p27kip1 [23] [24] [25].

NR4A1 has been found both to promote and inhibit tumorigenesis [26]. On one hand, NR4A1 behaves as a tumor suppressor by inhibiting growth of pancreatic cancer cells [27], and a double knock-out of NR4A1 and NR4A3 in mice was found to lead to the development of acute myeloid leukemia (AML); consistently, low expression of NR4A1 and NR4A3 has frequently been found in human AML [28] [29]. On the other hand, NR4A1 is commonly overexpressed in lung cancer patients and correlates to poor prognosis [30], and it has been shown to confer a proliferative advantage to colon cancer cells as well as increasing the invasive behavior of breast cancer by enhancing TGFβ signaling [31] [32]. Despite the well-established role of NR4A1 in apoptosis, its overexpression has also been reported to protect cells from apoptosis [33]. It is possible that the opposing effects of NR4A1 expression in different cancers may be explained by differences in post-translational modifications of NR4A1 and thereby its subcellular localization. Other aspects of NR4A1 functionality that may contribute to tumor development is its ability to promote cell migration, invasion through by promoting MMP-9 expression, inflammation, repair of DNA double-strand breaks and VEGF-induced angiogenesis [34] [35] [36] [37]. Recently, yet another way that NR4A1 can impact tumorigenesis was discovered where NR4A1 regulate the expression of stemness-related genes Oct-4 and Nanog in gastric cancers cells [37].

In the present work, we elucidated the signaling mechanisms by which PDGF-BB promotes NR4A1 expression and the role of NR4A1 in PDGF-mediated responses and tumorigenicity.

## Materials and Methods

### Cell culture

NIH3T3 cells were cultured in Dulbecco’s modified Eagle’s medium (DMEM) with 10% bovine serum (BS). The human glioblastoma cell lines, U-105MG and U-251MG [38], were cultured in RPMI-1640 supplemented with 10% fetal bovine serum (FBS) and 25 mM L-glutamine. For serum-starvation, cells were washed once and incubated in medium containing 0.1% bovine serum albumin (BSA). Recombinant human PDGF-BB was generously provided by Amgen (Thousand Oaks, CA). The inhibitors JNK Inhibitor II and BAY 11-7082 were from Calbiochem (San Diego, CA), CI-1040 (PD184352) and BIX02189 were from Sellek, and MG132 was from Sigma-Aldrich. For selection, 1 mg/ml of the antibiotic G418 Sulfate from Calbiochem (San Diego, CA) was added to the growth medium.

### Transfections

Downregulation of NR4A1 and Erk5 was performed by using 80 nM of specific Silencer Selected pre designed for NR4A1 (UUUCUGUACUGUGCGCUUGaa and UACCCGUCCAUGAAGGUGCtg) or for Erk5, both purchased from Ambion Life Technology. For every experiment performed, non targeting siRNA (stealth RNAi negative control Invitrogen #12935112) was used as a control. Transfection of siRNA was done with SilentFect from BioRad according to the manufacturer’s instructions. Levels of knockdown were analyzed after 48 h by immunoblot or by qPCR.

Three µl polyethyleneimine (2.5 mg/ml) diluted in 100 µl of serum-free DMEM was added to 2 µg plasmid DNA (Myc-DKK-Tagged ORF clone of mouse NR4A1 (MR209316) or TrueClone Pcmv6-Kan/Neo Vector from Origene), diluted in 100 µl of medium. The transfection mixture was incubated at room temperature for 20 min. Cells were washed and the medium was replaced with 2 ml/well of complete medium. The transfection mixture (200 µl) was added into the cell culture dish to give a total volume of 1.2 ml/well. For stable transfection, culture medium was replaced with selection medium containing 1 mg/ml G-418, after 48 h and culturing continued for one week.

### Real time PCR

Total DNA-free cellular RNA was extracted from cells treated with PDGF-BB for indicated periods of time with the RNeasy kit (Qiagen), and was reverse-transcribed (SuperScript II RNase; Invitrogen) to create cDNA templates. The PCR was performed by the CFX Manager (Bio-Rad) according to the manufacturer’s instructions. Glyceraldehyde-3-phosphate dehydrogenase was used as an endogenous control for the relative quantification of the target message. Specific primers were as follows: NR4A1, CTCGCCATCTACACCCAACT (forward) and CTTAGGCAACTGCCTCTGTCC (reverse); glyceraldehyde-3-phosphate dehydrogenase, CCCTTCATTGACCTCCACTACAT (forward) and GGGATTTCCATTGATGACAAG (reverse).

### Immunoblotting

Subconfluent cells were starved and incubated with vehicle or inhibitors at the indicated concentrations and thereafter stimulated with PDGF-BB (20 ng/ml, or as specified) for the indicated periods of time. Cells were washed two times in ice-cold phosphate-buffered saline and lysed in 20 mM Tris, pH 7.4, 150 mM NaCl, 5 mM EDTA, 1% Triton X-100, 0.1% SDS, 1% deoxycholate, 1 mM Pefa Bloc and 1 mM sodium orthovanadate. Extracts were clarified by centrifugation, and protein concentration was determined by the BCA protein assay (Pierce). Equal amounts of lysates were boiled with SDS sample buffer containing dithiothreitol. Proteins were separated by SDS-PAGE and then electro-transferred to polyvinylidene difluoride membranes (Immobilon P), which were blocked in 5% BSA or 5% milk in Tris-buffered saline solution containing 0.1% Tween-20. Primary antibodies were diluted according to the manufacturer’s instructions and membranes incubated overnight at 4°C. After washing, the membranes were incubated with horseradish peroxidase-conjugated anti-rabbit or anti-mouse IgG antibodies (both from Amersham Biosciences), and proteins were visualized using ECL immunoblotting detection systems from Roche Applied Science on a cooled charge-coupled device (CCD) camera (Bio-Rad). Densitometrical analysis of the immunoblots was performed using Quantity One software (Bio-Rad).

Mouse NR4A1 antibody (#554088) was purchased from BD Biosciences. Antibodies against human NR4A1 (#3960), phosphorylated Erk1/2 (#9106), phosphorylated AKT (#9271), phosphorylated Erk5 (#3371) and total Erk5 (#3372) were purchased from Cell Signaling Technology (Beverly, MA). α-Tubulin antibody was purchased from Sigma (St. Louis, MI). A rabbit antiserum recognizing Erk was raised against a peptide corresponding to the carboxyl-terminal sequence EETARFQPGYRS conjugated to KLH. A rabbit polyclonal antiserum against PDGFRβ was raised against a glutathione S-transferase fusion protein containing the COOH-terminal amino acid residues of PDGFRβ [39].

### Preparation of cytoplasmic and nuclear fractions

Cells were washed with ice-cold PBS twice, scraped in lysis buffer containing 10 mM MES, pH 6.2, 1.5 mM MgCl_2_, 10 mM NaCl, 1 mM EDTA, 5 mM dithiothreitol, and 1% Triton X-100, supplemented with protease inhibitors (1 mM Pefa Bloc, 1% Trasylol, 1 mM sodium orthovanadate). After centrifugation at 3000×g, the supernatant was collected as the cytoplasmic fraction. The pellet, enriched in nuclear proteins, was washed twice in lysis buffer supplemented with 1% NP-40 and then lysed in buffer containing 0.5% Triton X-100, 25 mM Tris-HCl, pH 10.5, 1 mM EDTA, 0.5 M NaCl, 5 mM β-mercaptoethanol, and supplemented with protease inhibitors. The supernatants from the two fractions were collected by centrifugation at 15,000×g for 30 min.

### Cell viability assay

Proliferation was evaluated in NIH3T3 cells. Ten thousand cells were plated in 96-well plates, serum-starved overnight and treated as indicated for 24 h. The assay was performed using CellTiter 96 AQueous One Solution Cell Proliferation Assay (Promega) according to manufacturer’s instructions. 20 µl/well of the CellTiter 96 AQueous One Solution Reagent containing tetrazolium compound [3-(4,5-dimethylthiazol-2-yl)-5-(3-carboxymethoxyphenyl)-2-(4-sulfophenyl)-2H-tetrazolium, inner salt; MTS] and an electron coupling reagent (phenazine ethosulfate; PES) were added to 100 µl of medium. The MTS tetrazolium compound is bioreduced by metabolically active cells into a colored formazan product that is soluble in tissue culture medium. After 3 h at 37°C in a humidified 5% CO_2_ atmosphere, the absorbance at 490 nm was recorded with an ELISA plate reader.

MTS proliferation assays were validated by manual counting of cells visualised with proliferation marker Ki-67 and nuclear marker DAPI. NIH3T3 cells grown on glass cover slips were fixed with washed in PBS, fixed in 100% acetone for 10 minutes. The cells were then incubated with Protein block Serum free solution (Dako) for 2 h. The cover slips were incubated overnight at 4°C in a primary rabbit anti-Ki-67 antibody solution (Cell Signaling; 1∶400), washed and incubated with a secondary Alexa-Fluor594 donkey anti-rabbit antibody (Life technology; 1∶1000) for 1 h at room temperature. After washing cover slips were mounted in DAPI-containing mountant (Vector Laboratories) and fluorescence was visualized under a microscope.

### Apoptosis assay

Subconfluent cell cultures were serum-starved and then incubated, in triplicates, for 48 h with or without 20 ng/ml of PDGF-BB; cells cultured in growth media were used as negative control. Cells were harvested, and the extent of apoptosis was determined by quantification of nucleosomes released into the cytoplasm using the Cell Death Detection ELISA Plus kit (Roche Applied Science) according to the manufacturer’s instructions.

### Cell migration assays

Ninety six-well ChemoTX (Neuroprobe, Gaithersburg, MD) cell migration microplate filters were coated with 50 µg/ml human fibronectin (#354008, BD Biosciences) for 1 h at room temperature. Cells were transfected with control or NR4A1 siRNA for 48 h, serum-starved overnight and then trypsinized into single cells. The wells of the ChemoTX microplate were filled with DMEM containing the indicated PDGF-BB concentrations. The filters were placed over the wells and 5×10^4^ cells were added on top of each filter. The chamber was incubated for 4 h at 37°C, 5% CO_2_. Non-migratory cells on the upper membrane surface were mechanically removed, and cells that had moved through the pores and adhered to the bottom of the filter were fixed by 3 min incubation in 96% ethanol, Giemsa (Sigma) stained and quantified by a CCD camera (Fuji). Quantifications were performed using Quantity One software.

### Soft agar colony formation assay

The bottom layer of a 6-well plate was prepared by pouring 800 µL of medium containing 0.8% low-melting-temperature agarose (Seaplaque), 3% serum, 100 µg/ml penicillin and 100 µg/ml streptomycin into each well, whereafter the agar was allowed to solidify. Cells were trypsinized and 2×10^4^ cells/ml were resuspended in medium containing 0.3% low-melting-temperature agarose, 3% serum, 100 µg/ml penicillin and streptomycin; 400 µL of this solution was poured as a top layer in each well. The cells were incubated for 5 days at 37°C, 5% CO_2_ in presence or absence of 50 ng/ml PDGF-BB and, after 10 days, the number and size of colonies were monitored manually using an Axiovert 40 CFL microscope.

## Results

### PDGF-BB induces NR4A1 expression through Erk1/2 and Erk5 MAP kinases and NF-κB

**Figure 1:**
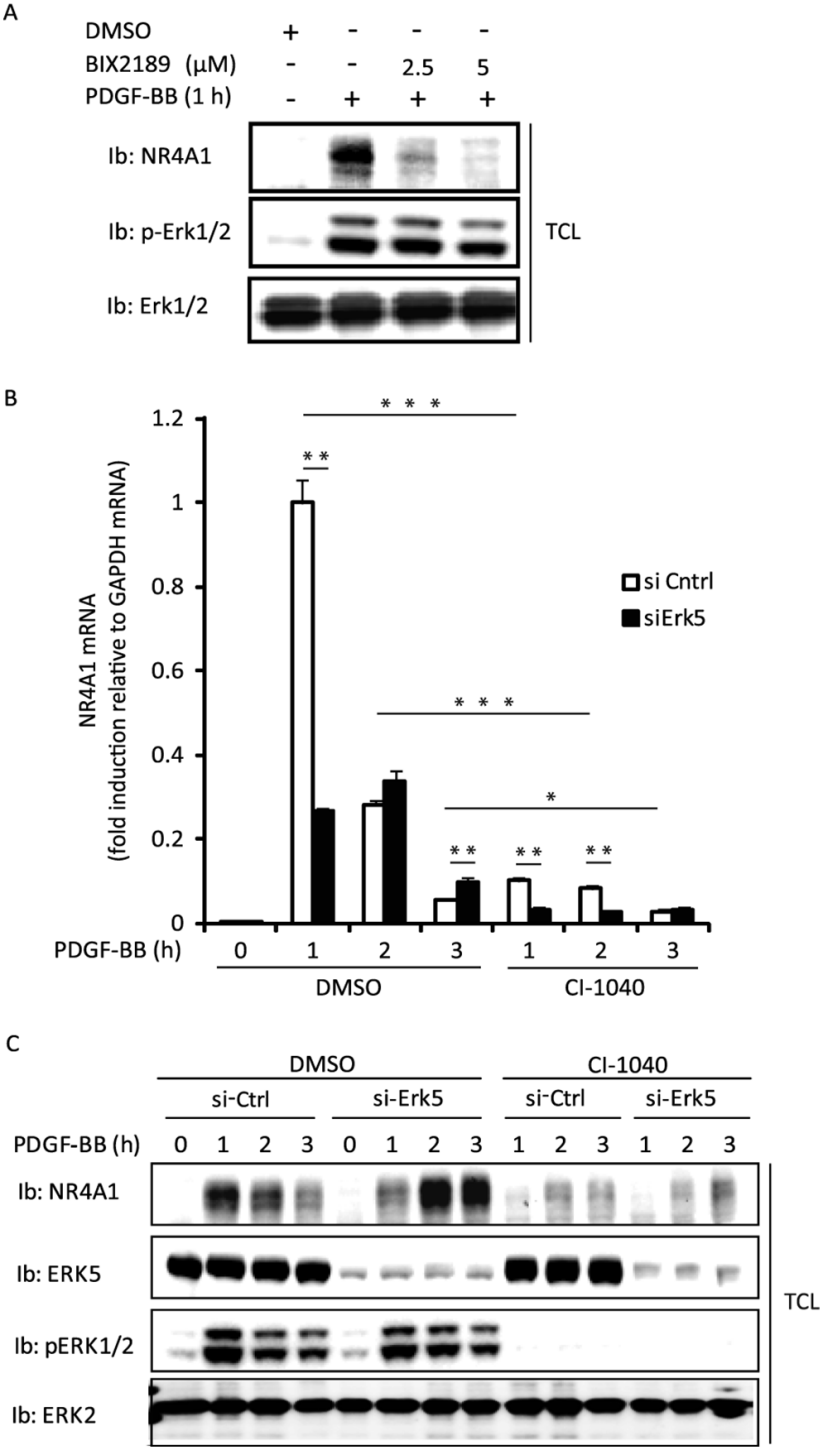
PDGF-BB induces NR4A1 via Erk1/2 and Erk5. NIH3T3 cells were serum-starved overnight in 0.1% BSA and treated with inhibitors starting 1 h before stimulation with PDGF-BB (20 ng/ml) for the indicated time periods. Total cell lysate (TCL) (A and C) were analyzed by immunoblotting (Ib) using NR4A1, Erk2, phospho-Erk1/2 and Erk5 antibodies. NR4A1 mRNA was measured with quantitative RT-PCR and panel B shows one out of three independent experiments performed; error bars indicate the standard deviation between three replicates. An asterisk (*) indicate a p-value≤0.05; with two (**) when it is ≤0.01 and with three (***) when it is ≤0.001.

Previous studies have implicated MAP-kinases in the regulation of NR4A1 expression [25] [40] [41], and we originally identified NR4A1 as a gene requiring Erk5 for its expression in response to PDGF-BB stimulation in a microarray analysis (unpublished data) comparing Erk5^−/−^ mouse embryonic fibroblasts transduced with empty virus or reconstituted with Erk5 [42]. To investigate the effect of the Erk5 MAP-kinase downstream of the PDGFR on the induction of NR4A1, we utilized the low molecular weight Erk5 inhibitor BIX02189. Treating cells with BIX02189 led to a decreased expression of NR4A1 after 1 h of PDGF-BB stimulation (Figure 1A). Since we saw a slight inhibition also of Erk1/2 phosphorylation, we also used a siRNA targeting Erk5; in cells where Erk5 was silenced, NR4A1 induction was delayed (Figure 1B and C). Inhibition of Erk1/2 MAP kinase by the MEK1/2 inhibitor CI-1040 repressed NR4A1 expression, and the combination of Erk5 siRNA and CI-1040 had the strongest effect with almost complete suppression of NR4A1 mRNA (Figure 1B). To verify that the changes observed on the mRNA level also translated to protein levels, we performed immunoblotting against NR4A1 under similar conditions. Inhibition of Erk1/2 signaling efficiently suppressed NR4A1 protein expression (Figure 1C). When Erk5 was silenced, PDGF-BB was still capable of promoting NR4A1 expression, however, with delayed kinetics. In contrast to the mRNA data, Erk5 silencing did not further enhance the CI-1040-induced suppression of NR4A1 protein expression.

**Figure 2:**
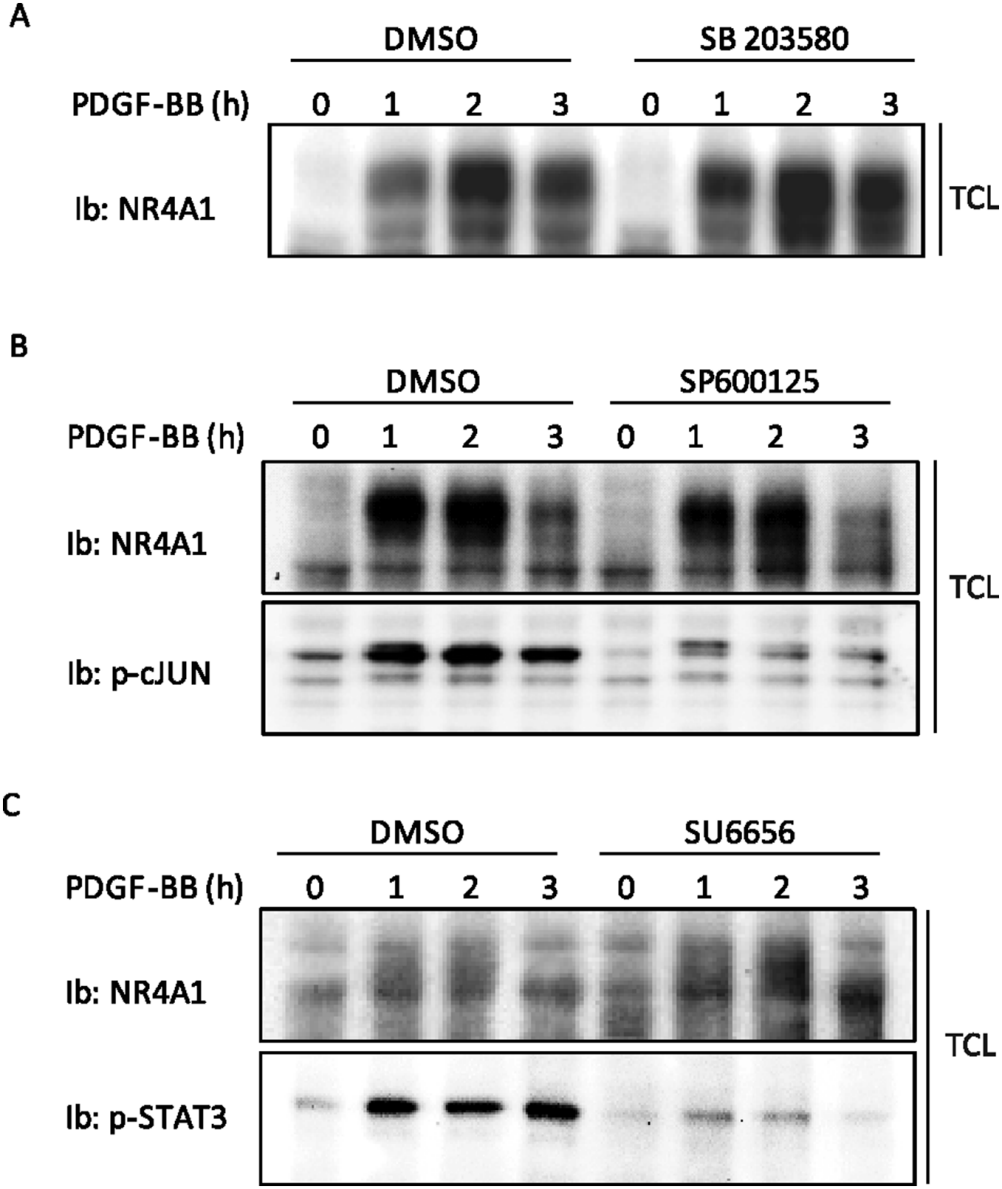
NR4A1 expression does not decrease after inhibition of p38, JNK and Src pathways. NIH3T3 cells were serum-starved overnight in 0.1% BSA and then pretreated for 1 h with DMSO or inhibitors SB203580 10 µM (A), SP600125 10 µM (B) and SU6656 2 µM (C), and then stimulated for indicated time periods with PDGF-BB (20 ng/ml); total cell lysates (TCL) were prepared and subjected to immunoblotting (Ib).

Moreover, we analyzed NR4A1 expression in the presence of inhibitors targeting other major pathways downstream of the PDGFR. PDGF-BB-induced NR4A1 protein expression was not significantly decreased after inhibition of the MAP-kinase p38, by SB203580 (**Figure 2A**), the MAP-kinase JNK, by SP600125, (**Figure 2B**), or the Src kinase by SU6656 (**Figure 2C**).

**Figure 3:**
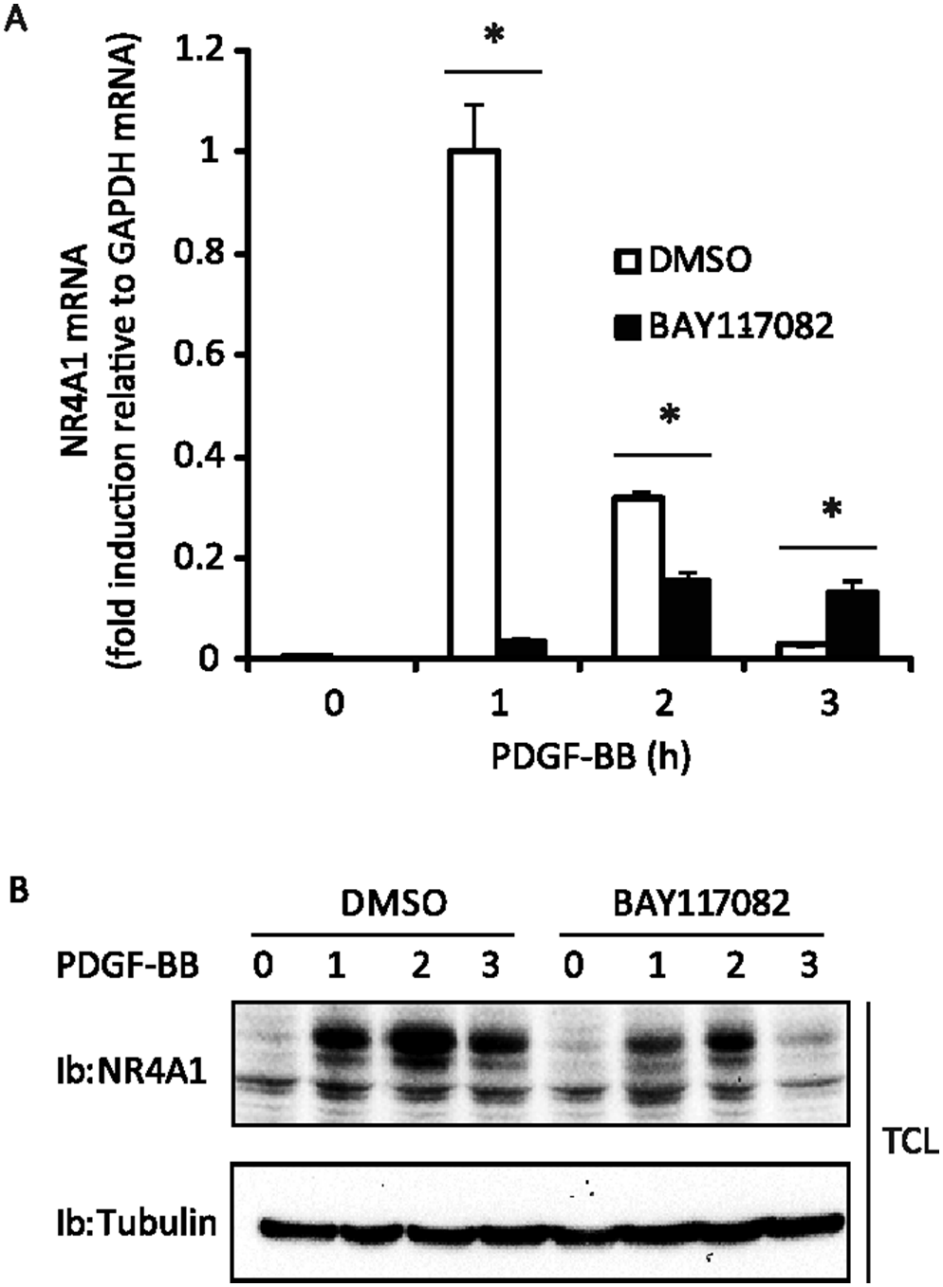
PDGF-BB-induced NR4A1 expression is decreased after treatment with the NF-κB inhibitor BAY11-7082. NIH3T3 cells were serum-starved overnight in 0.1% BSA. Cells were pretreated for 4 h with DMSO or NF-κB inhibitor BAY11-7082 (10 µM) (A and B) and then stimulated for indicated time periods with PDGF-BB (20 ng/ml). mRNA was measured with quantitative RT-PCR (A) and total cell lysate (TCL) analyzed by immunoblotting (Ib) using NR4A1 antibody and tubulin, as loading control (B). Panel A shows the result of one out of three independent experiments performed; error bars indicate the standard deviation between three replicates. An asterisk (*) indicate a p-value≤0.05.

In some experimental systems, it has been observed that NR4A1 expression results in an increased IκB expression and hence termination of NF-κB signaling. To explore the possibility that, conversely, NF-κB affects NR4A1 expression, we treated cells with an inhibitor of the NF-κB pathway (BAY11-7082), and analyzed the effect on PDGF-BB-induced NR4A1 mRNA and protein levels. We found that upon NF-κB inhibition both NR4A1 mRNA and protein levels were suppressed (**Figure 3A and B**, respectively). We further treated cells with the proteasomal inhibitor MG132 that blocks the degradation of, among other things, the NF-κB inhibitor protein IκB, and measured PDGF-BB-induced NR4A1. A strong suppression of both NR4A1 mRNA and protein levels was seen after proteasomal inhibition (Data not shown).

In summary, Erk5, Erk1/2 and NF-κB contributed to PDGF-BB-induced increase in NR4A1 expression, whereas inhibition of other kinases activated by PDGFR, i.e. p38, JNK and Src, did not decrease NR4A1 protein expression.

### NR4A1 localizes to both cytoplasm and nucleus in PDGF-BB stimulated cells

**Figure 4:**
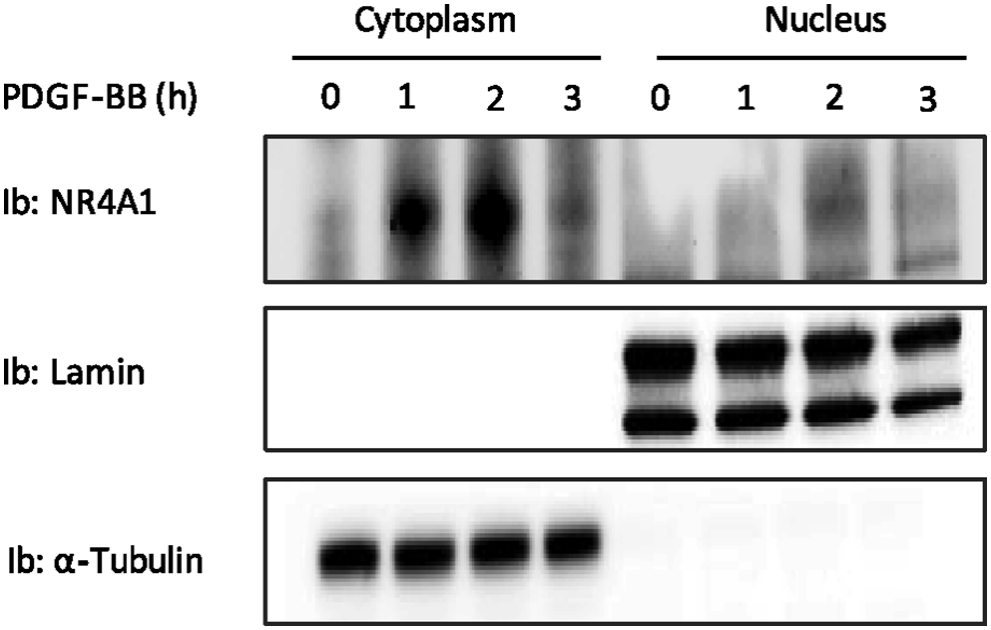
A small pool of the cytoplasmic NR4A1 translocates into the nucleus after long PDGF-BB stimulation. Cytoplasmic and nuclear fractions were prepared from NIH3T3 cells serum-starved overnight in 0.1% BSA and stimulated with PDGF-BB (20 ng/ml) for indicated periods of time and NR4A1 levels were analyzed by immunoblotting (Ib). The purity of the fraction were confirmed by immunoblotting for the nuclear marker Lamin and the cytoplasmic marker Tubulin.

It has been shown that the function of NR4A1 depends on its subcellular localization; nuclear NR4A1 regulates cell proliferation, whereas cytoplasmic NR4A1 affects survival [9] [43] [44]. To elucidate the localization of NR4A1 in cells treated with PDGF-BB, we performed biochemical nuclear and cytoplasmic fractionation and subjected the samples to immunoblotting for NR4A1. We found that the majority of NR4A1 appeared in the cytoplasm, whereas a fraction was translocated to the nucleus after continued PDGF-BB treatment (**Figure 4**). The observation that in PDGF-BB-stimulated NIH3T3 cells NR4A1 is localized both in the cytoplasmic and in the nuclear compartments suggests a complex regulation of the proliferation-apoptosis balance and that integration of other pathways may determine the final outcome.

### NR4A1 knock-down in NIH3T3 cells increases PDGF-BB-induced proliferation without affecting survival or migration

**Figure 5:**
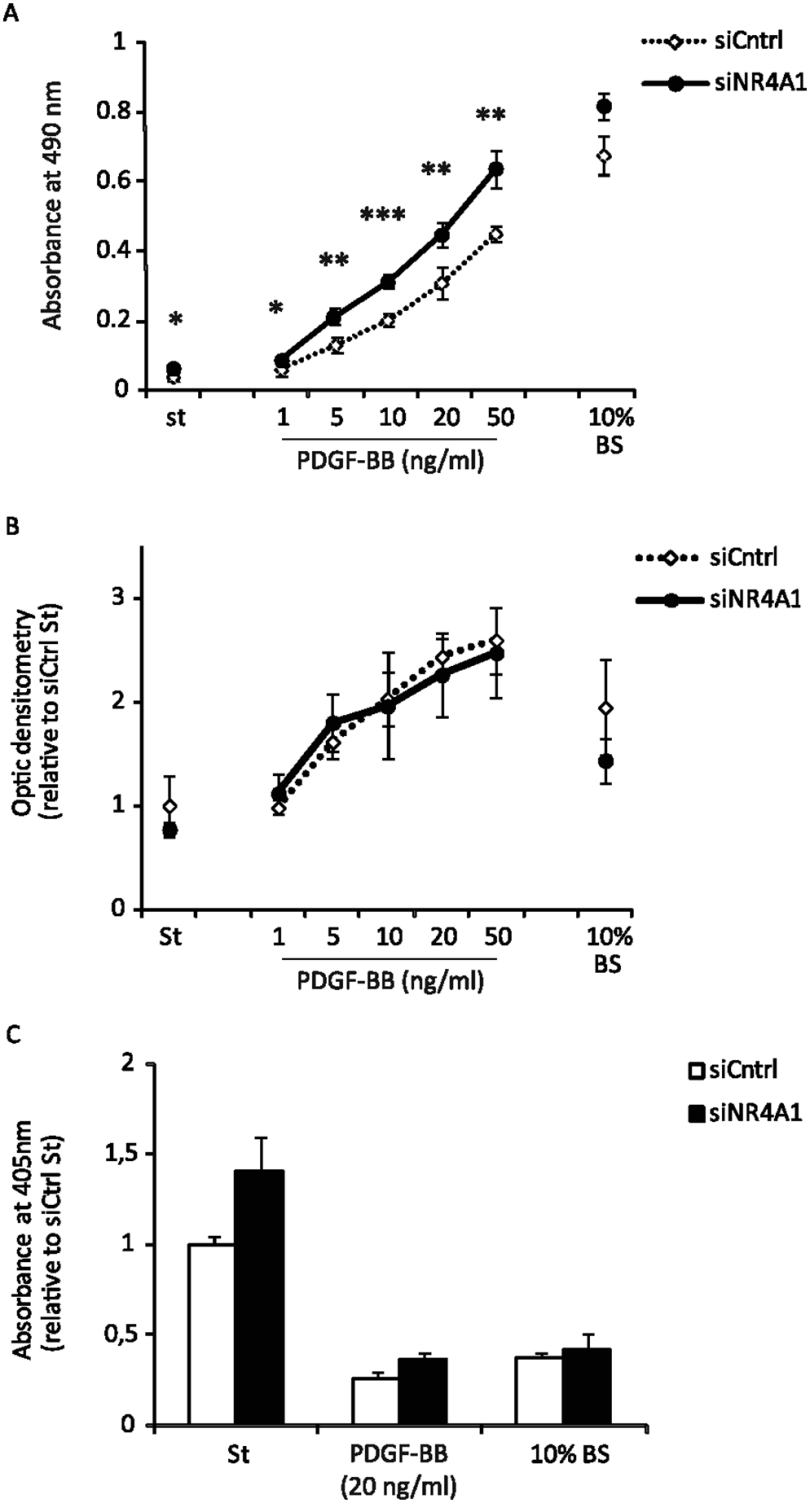
Knock-down of NR4A1 increases PDGF-BB-induced proliferation of NIH3T3 cells without affecting survival or migration. NIH3T3 cells were transfected with siRNA against NR4A1 or control siRNA, serum-starved over-night and then stimulated with indicated concentrations of PDGF-BB. Cell proliferation was determined in three independent experiments by an MTS assay after 24 h of indicated treatments (A). A representative experiment is shown and error bars indicate the standard deviation between four replicates and it has been indicated with an asterisk (*) when the p-value is ≤0.05; with two (**) when it is ≤0.01 and with three (***) when it is ≤0.001. The chemotaxis promoted by 4 h stimulation with the indicated PDGF-BB concentrations was measured by Giemsa staining and quantification of the cells that migrated across the filter (B); error bars indicate the standard deviation between four replicates of a representative experiment, repeated independently three times. The apoptosis was determined by ELISA assay measuring nucleosomes released into the cytoplasm in three independent experiments each performed in duplicates and analyzed together; the error bar represent the standard error of the mean (C).

Previous reports have indicated roles for NR4A1 in survival, proliferation and cell migration [23] [45] [46]. To investigate the functional role of NR4A1 in PDGF-BB-stimulated cells, we silenced NR4A1 expression and performed functional assays. We observed that cells depleted of NR4A1 display an increased proliferation rate measured by MTS assay (**Figure 5A**). To rule out the possibility that the difference observed in the MTS assay was due to an increased metabolic activity, we validated the data by immunofluorescence; silencing of NR4A1 led to an increased number of cells positive for the proliferation marker Ki-67 (data not shown).

To elucidate the chemotactic response to PDGF-BB, we evaluated transwell migration of NR4A1 knock-down cells. We did not find any significant change in chemotaxis toward PDGF-BB in cells depleted of NR4A1 compared to control cells (**Figure 5B**).

There are reports claiming that NR4A1 may interact with proteins in the mitochondria and thereby promote apoptosis [15] [16] [17] [47], however, we did not observe any change in the ability of PDGF-BB to protect cells from serum starvation-induced apoptosis when comparing control cells to those with reduced NR4A1 expression (**Figure 5C**). In addition, we did not find any alteration of apoptosis in the serum-starved cells depleted of NR4A1 in the absence of PDGF-BB.

### NR4A1 expression is important for the ability of PDGF-BB to promote anchorage-independent growth in soft agar

**Figure 6:**
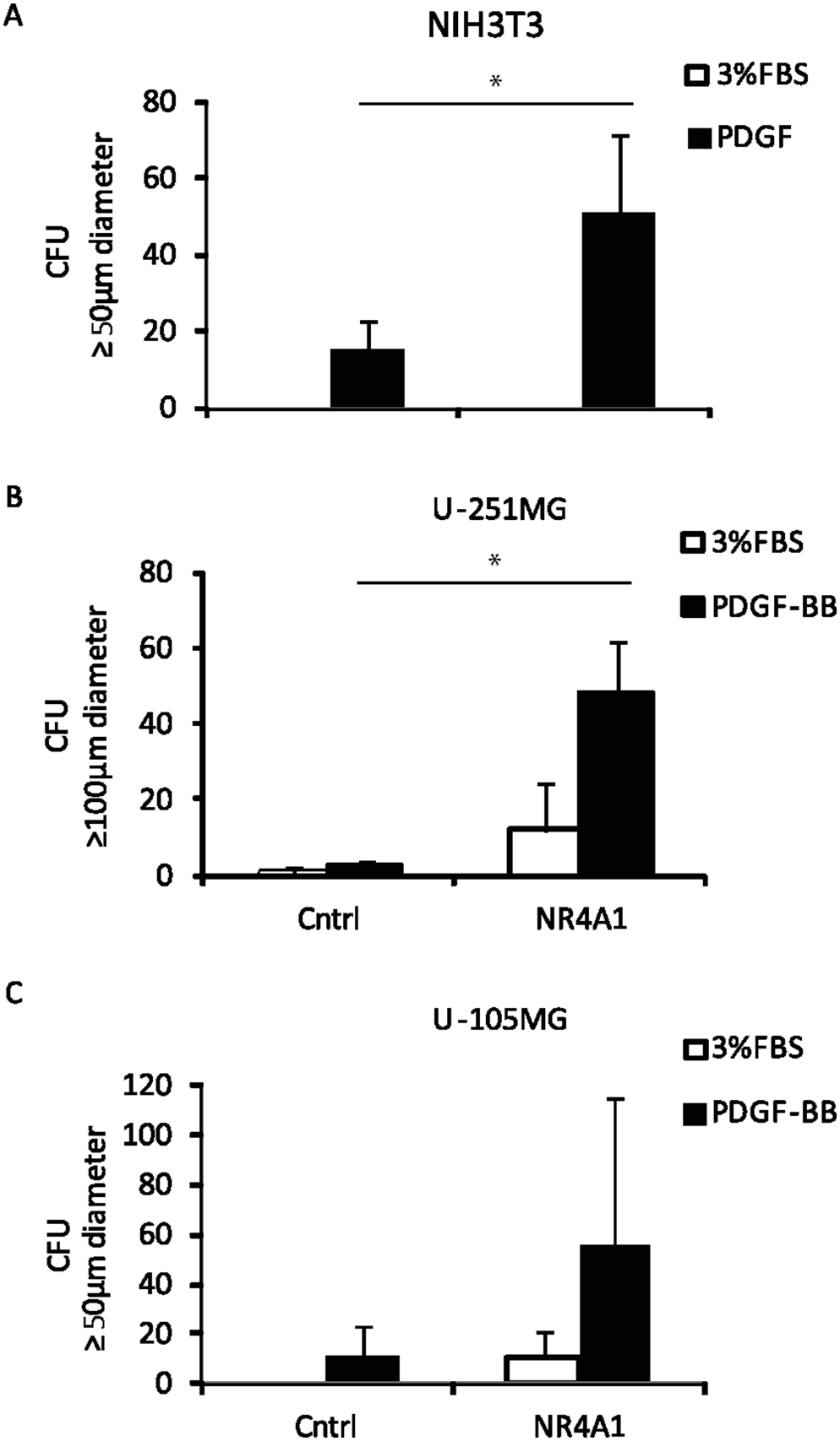
NR4A1 promotes PDGF-BB-induced anchorage-independent growth. NR4A1 was overexpressed by stable transfection in NIH3T3 cells (A), and U-251MG (B) and U-105MG (C) glioblastoma cells; cells were cultured in soft agar in starvation medium (3% serum) with or without 50 ng/ml PDGF-BB. The number and size of the colony forming unit (CFU) were measured after 10 days. For each cell line a representative experiment is shown, out of at least two independently performed, and the standard deviation between three replicates is indicated in the error bar. A p-value≤0.05 is indicated with an asterisk (*).

NR4A1 has been found to be overexpressed in many tumors. We therefore evaluated the role of NR4A1 for *in vitro* tumorigenicity, i.e. colony formation in soft agar. NIH3T3 cells do not form colonies in soft agar in the absence of PDGF-BB, however, PDGF-BB stimulation induced colony formation in soft agar and the effect was enhanced after overexpression of NR4A1 (Figure 6A). A strong enhancement of PDGF-BB stimulated colony growth was seen by overexpression of NR4A1 also in the glioblastoma cell line U-251MG (Figure 6B) and U-105MG exhibits a similar trend (Figure 6C). The U-251MG cells formed large colonies in soft agar, therefore we reported only those with a diameter larger than 100 µm.

## Discussion

In the present study, we found that PDGF-BB stimulation of NIH3T3 cells resulted in a robust upregulation of NR4A1 mRNA and protein. In addition, the other two NR4A family members NR4A2 and NR4A3 were also upregulated, at least at the mRNA level (**Figure S1**). Furthermore, we found that NR4A1 expression relied to a large extent on Erk1/2 and NF-κB signaling, whereas Erk5 contributed to the rapid kinetics of upregulation. Initially the newly synthesized NR4A1 had a cytoplasmic localization, whereas prolonged PDGF-BB treatment dispatched part of the NR4A1 population to the nucleus. Functionally, we observed that NR4A1 downregulation increased proliferation promoted by mitogenic factors, such as PDGF-BB and serum, both using MTS assay and Ki-67 expression, whereas no effects on chemotaxis or apoptosis of NIH3T3 fibroblasts were observed. However, PDGF-BB-driven soft agar colony formation of NIH3T3 and glioma cells was strongly enhanced by NR4A1 expression.

The central role of Erk1/2 and NF-κB for NR4A1 induction that emerged from our work is consistent with previous studies that implicated MAP-kinases or NF-κB signaling in regulation of expression of NR4A family members [25] [40] [41] [48] [49] [50] [51]. It is possible that activation of PDGFRβ, via activation of Erk1/2 and Erk5, promotes the nuclear localization of NF-κB, which increases the transcription of NR4A1. The finding that in amyloid plaques Erk1/2 is activated and directly interacts with IκB kinase promoting NF-κB activation [52], is consistent with this possibility.

Consistent with studies in smooth muscle cells [53] [54], we found that NR4A1 reduces proliferation. However, in other studies, e.g. using endothelial cells and lung cancer cells, NR4A1 promotes cell proliferation [34] [25] [30]. It is possible that the different effects of NR4A1 on cell proliferation are related to the balance between nuclear and cytoplasmic localization of NR4A1 which may influence its function, as well as the presence of other pathways integrating with NR4A1 in regulating these processes.

NR4A1 promotes mesenchymal stromal cell migration [55]. Since PDGF-BB has a robust chemotactic effect in fibroblasts, we investigated whether manipulation of NR4A1 expression affected cell migration. We did not see any effect of NR4A1 silencing on PDGF-BB-induced cell migration. Similarly, we did not observe any NR4A1-dependence in the ability of PDGF-BB to protect cells against serum-induced apoptosis, despite several studies pointing to a critical role for NR4A1 in regulating cell survival [56] [57]. One possible reason for the lack of effect in NIH3T3 cells is redundancy with the two other NR4A family members NR4A2 and NR4A3, both of which, at least at the mRNA level, are induced by PDGF-BB stimulation in NIH3T3 cells (**Figure S1**), and may overlap in function with NR4A1.

Overexpression of NR4A1 has been seen in several types of tumors and NR4A1 overexpression can protect cells from apoptosis [17] [56]. The effect of NR4A1 on apoptosis is context-dependent since in other systems, such as TCR-mediated apoptosis, NR4A1 promotes this process [18] [19]. It is possible that the subcellular localization of NR4A1 impacts its effect on cell survival. Consistent with the observation that NR4A1 is overexpressed in tumor cells, we show that NR4A1 expression is essential for glioblastoma cell colony formation in soft agar. This is consistent with the observation that in different tumor types, including glioblastoma, migration and invasion are associated with Erk1/2 activation and NR4A1 expression [58] [59].

This study clarifies the regulation of NR4A1 in NIH3T3 and demonstrates its role in PDGF-BB-meditated cell transformation both in NIH3T3 and in glioblastoma-derived cell lines; our findings suggest that NR4A1 may be a target in cancer treatment.

## Supporting Information

Figure S1 **NR4A2 (Nurr1) and NR4A3 (NOR-1) mRNA are induced by PDGF-BB.** NIH3T3 cells were treated with Erk5 siRNA or control siRNA and then stimulated by PDGF-BB (20 ng/ml) for indicated time periods. NR4A2 (A) and NR4A3 (B) mRNA levels were measured by quantitative RT-PCR.
(TIF)

## References

[1] Heldin CH, Westermark B (1999) Mechanism of action and in vivo role of platelet-derived growth factor. Physiol Rev 79: 1283–1316. 10508235 10.1152/physrev.1999.79.4.1283

[2] Fredriksson L, Li H, Eriksson U (2004) The PDGF family: four gene products form five dimeric isoforms. Cytokine Growth Factor Rev 15: 197–204 10.1016/j.cytogfr.2004.03.007 S1359610104000176 [pii] 15207811

[3] Ostman A, Heldin CH (2007) PDGF receptors as targets in tumor treatment. Adv Cancer Res 97: 247–274 10.1016/S0065-230X(06)97011-0 17419949

[4] Keshet Y, Seger R (2010) The MAP kinase signaling cascades: a system of hundreds of components regulates a diverse array of physiological functions. Methods Mol Biol 661: 3–38 10.1007/978-1-60761-795-21 20811974

[5] Cargnello M, Roux PP (2011) Activation and function of the MAPKs and their substrates, the MAPK-activated protein kinases. Microbiol Mol Biol Rev 75: 50–83 10.1128/MMBR.00031-10 21372320 PMC3063353

[6] Kondoh K, Terasawa K, Morimoto H, Nishida E (2006) Regulation of nuclear translocation of extracellular signal-regulated kinase 5 by active nuclear import and export mechanisms. Mol Cell Biol 26: 1679–1690 10.1128/MCB.26.5.1679-1690.2006 16478989 PMC1430242

[7] Kasler HG, Victoria J, Duramad O, Winoto A (2000) ERK5 is a novel type of mitogen-activated protein kinase containing a transcriptional activation domain. Mol Cell Biol 20: 8382–8389. 11046135 10.1128/mcb.20.22.8382-8389.2000 PMC102145

[8] To SK, Zeng JZ, Wong AS (2012) Nur77: a potential therapeutic target in cancer. Expert Opin Ther Targets 16: 573–585 10.1517/14728222.2012.680958 22537097

[9] Wingate AD, Arthur JS (2006) Post-translational control of Nur77. Biochem Soc Trans 34: 1107–1109 10.1042/BST0341107 17073761

[10] Cheng LE, Chan FK, Cado D, Winoto A (1997) Functional redundancy of the Nur77 and Nor-1 orphan steroid receptors in T-cell apoptosis. EMBO J 16: 1865–1875 10.1093/emboj/16.8.1865 9155013 PMC1169790

[11] Han YH, Cao X, Lin B, Lin F, Kolluri SK, et al (2006) Regulation of Nur77 nuclear export by c-Jun N-terminal kinase and Akt. Oncogene 25: 2974–2986 10.1038/sj.onc.1209358 16434970

[12] Liu B, Wu JF, Zhan YY, Chen HZ, Zhang XY, et al (2007) Regulation of the orphan receptor TR3 nuclear functions by c-Jun N terminal kinase phosphorylation. Endocrinology 148: 34–44 10.1210/en.2006-0800 17023523

[13] Kang SA, Na H, Kang HJ, Kim SH, Lee MH, et al (2010) Regulation of Nur77 protein turnover through acetylation and deacetylation induced by p300 and HDAC1. Biochem Pharmacol 80: 867–873 10.1016/j.bcp.2010.04.026 20438716

[14] You B, Jiang YY, Chen S, Yan G, Sun J (2009) The orphan nuclear receptor Nur77 suppresses endothelial cell activation through induction of IkappaBalpha expression. Circ Res 104: 742–749 10.1161/CIRCRESAHA.108.192286 19213954

[15] Wilson AJ, Arango D, Mariadason JM, Heerdt BG, Augenlicht LH (2003) TR3/Nur77 in colon cancer cell apoptosis. Cancer Res 63: 5401–5407. 14500374

[16] Kolluri SK, Zhu X, Zhou X, Lin B, Chen Y, et al (2008) A short Nur77-derived peptide converts Bcl-2 from a protector to a killer. Cancer Cell 14: 285–298 10.1016/j.ccr.2008.09.002 18835031 PMC2667967

[17] Thompson J, Winoto A (2008) During negative selection, Nur77 family proteins translocate to mitochondria where they associate with Bcl-2 and expose its proapoptotic BH3 domain. J Exp Med 205: 1029–1036 10.1084/jem.20080101 18443228 PMC2373836

[18] Liu ZG, Smith SW, McLaughlin KA, Schwartz LM, Osborne BA (1994) Apoptotic signals delivered through the T-cell receptor of a T-cell hybrid require the immediate-early gene nur77. Nature 367: 281–284 10.1038/367281a0 8121494

[19] Woronicz JD, Calnan B, Ngo V, Winoto A (1994) Requirement for the orphan steroid receptor Nur77 in apoptosis of T-cell hybridomas. Nature 367: 277–281 10.1038/367277a0 8121493

[20] Pearen MA, Muscat GE (2010) Minireview: Nuclear hormone receptor 4A signaling: implications for metabolic disease. Mol Endocrinol 24: 1891–1903 10.1210/me.2010-0015 20392876 PMC5417389

[21] Stocco CO, Zhong L, Sugimoto Y, Ichikawa A, Lau LF, et al (2000) Prostaglandin F2alpha-induced expression of 20alpha-hydroxysteroid dehydrogenase involves the transcription factor NUR77. J Biol Chem 275: 37202–37211 10.1074/jbc.M006016200 M006016200 [pii] 10973968

[22] Martin LJ, Tremblay JJ (2005) The human 3beta-hydroxysteroid dehydrogenase/Delta5-Delta4 isomerase type 2 promoter is a novel target for the immediate early orphan nuclear receptor Nur77 in steroidogenic cells. Endocrinology 146: 861–869 10.1210/en.2004-0859 15498889

[23] Arkenbout EK, van Bragt M, Eldering E, van Bree C, Grimbergen JM, et al (2003) TR3 orphan receptor is expressed in vascular endothelial cells and mediates cell cycle arrest. Arter Thromb Vasc Biol 23: 1535–1540 10.1161/01.ATV.0000084639.16462.7A 01.ATV.0000084639.16462.7A [pii] 12842839

[24] Arkenbout EK, Dekker RJ, de Vries CJ, Horrevoets AJ, Pannekoek H (2003) Focusing on transcription factor families in atherogenesis: the function of LKLF and TR3. Thromb Haemost 89: 522–529 10.1267/THRO0303052203030522pii 12624637

[25] Wang L, Gong F, Dong X, Zhou W, Zeng Q (2010) Regulation of vascular smooth muscle cell proliferation by nuclear orphan receptor Nur77. Mol Cell Biochem 341: 159–166 10.1007/s11010-010-0447-0 20411306

[26] Safe S, Jin U-H, Hedrick E, Reeder A, Lee S-O (2014) Minireview: role of orphan nuclear receptors in cancer and potential as drug targets. Mol Endocrinol Baltim Md 28: 157–172 10.1210/me.2013-1291 PMC3896638 24295738

[27] Lee SO, Abdelrahim M, Yoon K, Chintharlapalli S, Papineni S, et al (2010) Inactivation of the orphan nuclear receptor TR3/Nur77 inhibits pancreatic cancer cell and tumor growth. Cancer Res 70: 6824–6836 10.1158/0008-5472.CAN-10-1992 20660371 PMC2988472

[28] Deutsch AJ, Angerer H, Fuchs TE, Neumeister P (n.d.) The nuclear orphan receptors NR4A as therapeutic target in cancer therapy. Anticancer Agents Med Chem 12: 1001–1014 doi:ACAMC-EPUB-20120502-001 [pii] 10.2174/187152012803529619 22583411

[29] Mullican SE, Zhang S, Konopleva M, Ruvolo V, Andreeff M, et al (2007) Abrogation of nuclear receptors Nr4a3 and Nr4a1 leads to development of acute myeloid leukemia. Nat Med 13: 730–735 10.1038/nm1579 17515897

[30] Kolluri SK, Bruey-Sedano N, Cao X, Lin B, Lin F, et al (2003) Mitogenic effect of orphan receptor TR3 and its regulation by MEKK1 in lung cancer cells. Mol Cell Biol 23: 8651–8667. 14612408 10.1128/MCB.23.23.8651-8667.2003 PMC262666

[31] Wu H, Lin Y, Li W, Sun Z, Gao W, et al. (n.d.) Regulation of Nur77 expression by beta-catenin and its mitogenic effect in colon cancer cells. FASEB J 25: 192–205 10.1096/fj.10-166462 PMC3005431 20847229

[32] Zhou F, Drabsch Y, Dekker TJA, de Vinuesa AG, Li Y, et al (2014) Nuclear receptor NR4A1 promotes breast cancer invasion and metastasis by activating TGF-β signalling. Nat Commun 5: 3388 10.1038/ncomms4388 24584437

[33] De Léséleuc L, Denis F (2005) Inhibition of apoptosis by Nur77 through NF-κB activity modulation. Cell Death Differ 13: 293–300 10.1038/sj.cdd.4401737 16082387

[34] Zeng H, Qin L, Zhao D, Tan X, Manseau EJ, et al (2006) Orphan nuclear receptor TR3/Nur77 regulates VEGF-A-induced angiogenesis through its transcriptional activity. J Exp Med 203: 719–729 10.1084/jem.20051523 16520388 PMC2118245

[35] Zhao D, Qin L, Bourbon PM, James L, Dvorak HF, et al (2011) Orphan nuclear transcription factor TR3/Nur77 regulates microvessel permeability by targeting endothelial nitric oxide synthase and destabilizing endothelial junctions. Proc Natl Acad Sci U A 108: 12066–12071 10.1073/pnas.1018438108 PMC3142008 21730126

[36] Zhao BX, Chen HZ, Du XD, Luo J, He JP, et al (2011) Orphan receptor TR3 enhances p53 transactivation and represses DNA double-strand break repair in hepatoma cells under ionizing radiation. Mol Endocrinol 25: 1337–1350 10.1210/me.2011-0081 21659476 PMC5417243

[37] Zhan Y, He J, Chen H, Wang W, Cai J (2013) Orphan receptor TR3 is essential for the maintenance of stem-like properties in gastric cancer cells. Cancer Lett 329: 37–44 10.1016/j.canlet.2012.09.022 23043761

[38] Pontén J, Macintyre EH (1968) Long term culture of normal and neoplastic human glia. Acta Pathol Microbiol Scand 74: 465–486. 4313504 10.1111/j.1699-0463.1968.tb03502.x

[39] Jurek A, Amagasaki K, Gembarska A, Heldin C-H, Lennartsson J (2009) Negative and positive regulation of MAPK phosphatase 3 controls platelet-derived growth factor-induced Erk activation. J Biol Chem 284: 4626–4634 10.1074/jbc.M808490200 19106095

[40] Sakaue M, Adachi H, Dawson M, Jetten AM (2001) Induction of Egr-1 expression by the retinoid AHPN in human lung carcinoma cells is dependent on activated ERK1/2. Cell Death Differ 8: 411–424 10.1038/sj.cdd.4400818 11550093

[41] Darragh J, Soloaga A, Beardmore VA, Wingate AD, Wiggin GR, et al (2005) MSKs are required for the transcription of the nuclear orphan receptors Nur77, Nurr1 and Nor1 downstream of MAPK signalling. Biochem J 390: 749–759 10.1042/BJ20050196 15910281 PMC1199668

[42] Sohn SJ, Li D, Lee LK, Winoto A (2005) Transcriptional Regulation of Tissue-Specific Genes by the ERK5 Mitogen-Activated Protein Kinase. Mol Cell Biol 25: 8553–8566 10.1128/MCB.25.19.8553-8566.2005 16166637 PMC1265748

[43] Wingate AD, Campbell DG, Peggie M, Arthur JS (2006) Nur77 is phosphorylated in cells by RSK in response to mitogenic stimulation. Biochem J 393: 715–724 10.1042/BJ20050967 16223362 PMC1360724

[44] Zhang XK (2007) Targeting Nur77 translocation. Expert Opin Ther Targets 11: 69–79 10.1517/14728222.11.1.69 17150035

[45] Alexopoulou AN, Leao M, Caballero OL, Da Silva L, Reid L, et al (2010) Dissecting the transcriptional networks underlying breast cancer: NR4A1 reduces the migration of normal and breast cancer cell lines. Breast Cancer Res 12: R51 10.1186/bcr2610 20642837 PMC2949640

[46] Yoon K, Lee SO, Cho SD, Kim K, Khan S, et al (2011) Activation of nuclear TR3 (NR4A1) by a diindolylmethane analog induces apoptosis and proapoptotic genes in pancreatic cancer cells and tumors. Carcinogenesis 32: 836–842 10.1093/carcin/bgr040 21362629 PMC3106434

[47] Wu Q, Liu S, Ye XF, Huang ZW, Su WJ (2002) Dual roles of Nur77 in selective regulation of apoptosis and cell cycle by TPA and ATRA in gastric cancer cells. Carcinogenesis 23: 1583–1592. 12376465 10.1093/carcin/23.10.1583

[48] Kovalovsky D, Refojo D, Liberman AC, Hochbaum D, Pereda MP, et al (2002) Activation and induction of NUR77/NURR1 in corticotrophs by CRH/cAMP: involvement of calcium, protein kinase A, and MAPK pathways. Mol Endocrinol 16: 1638–1651. 12089357 10.1210/mend.16.7.0863

[49] McEvoy AN, Murphy EA, Ponnio T, Conneely OM, Bresnihan B, et al (2002) Activation of nuclear orphan receptor NURR1 transcription by NF-kappa B and cyclic adenosine 5′-monophosphate response element-binding protein in rheumatoid arthritis synovial tissue. J Immunol 168: 2979–2987. 11884470 10.4049/jimmunol.168.6.2979

[50] Pei L, Castrillo A, Chen M, Hoffmann A, Tontonoz P (2005) Induction of NR4A orphan nuclear receptor expression in macrophages in response to inflammatory stimuli. J Biol Chem 280: 29256–29262 10.1074/jbc.M502606200 15964844

[51] Smith AG, Lim W, Pearen M, Muscat GE, Sturm RA (2011) Regulation of NR4A nuclear receptor expression by oncogenic BRAF in melanoma cells. Pigment Cell Melanoma Res 24: 551–563 10.1111/j.1755-148X.2011.00843.x 21362156

[52] Wang X, Chen Q, Xing D (2012) Focal adhesion kinase activates NF-κB via the ERK1/2 and p38MAPK Pathways in amyloid-β25-35-induced apoptosis in PC12 cells. J Alzheimers Dis JAD 32: 77–94 10.3233/JAD-2012-120526 22776966

[53] Liu Y, Zhang J, Yi B, Chen M, Qi J, et al (2014) Nur77 suppresses pulmonary artery smooth muscle cell proliferation through inhibition of the STAT3/Pim-1/NFAT pathway. Am J Respir Cell Mol Biol 50: 379–388 10.1165/rcmb.2013-0198OC 24047441 PMC3930951

[54] Hinze AV, Mayer P, Harst A, Kügelgen I (2013) P2X1 receptor-mediated inhibition of the proliferation of human coronary smooth muscle cells involving the transcription factor NR4A1. Purinergic Signal 9: 677–686 10.1007/s11302-013-9380-5 23873636 PMC3889386

[55] Maijenburg MW, van der Schoot CE, Voermans C (2012) Mesenchymal stromal cell migration: possibilities to improve cellular therapy. Stem Cells Dev 21: 19–29 10.1089/scd.2011.0270 21732817

[56] Bras A, Albar JP, Leonardo E, de Buitrago GG, Martinez AC (2000) Ceramide-induced cell death is independent of the Fas/Fas ligand pathway and is prevented by Nur77 overexpression in A20 B cells. Cell Death Differ 7: 262–271 10.1038/sj.cdd.4400653 10745271

[57] Suzuki S, Suzuki N, Mirtsos C, Horacek T, Lye E, et al (2003) Nur77 as a survival factor in tumor necrosis factor signaling. Proc Natl Acad Sci U A 100: 8276–8280 10.1073/pnas.0932598100 0932598100 [pii] PMC166219 12815108

[58] Inaoka Y, Yazawa T, Uesaka M, Mizutani T, Yamada K, et al (2008) Regulation of NGFI-B/Nur77 gene expression in the rat ovary and in leydig tumor cells MA-10. Mol Reprod Dev 75: 931–939 10.1002/mrd.20788 18163434

[59] Li Z, Li C, Du L, Zhou Y, Wu W (2013) Human chorionic gonadotropin beta induces migration and invasion via activating ERK1/2 and MMP-2 in human prostate cancer DU145 cells. PLoS One 8: e54592 10.1371/journal.pone.0054592PONE-D-12-16732 PONE-D-12-16732 [pii] 23424616 PMC3570544

