# Supplementary figures and images for: NR4A1 Promotes PDGF-BB-Induced Cell Colony Formation in Soft Agar
Source: PLoS One. 2014 Sep 30;9(9):e109047. doi: 10.1371/journal.pone.0109047 (PMC4182636; doi:10.1371/journal.pone.0109047)

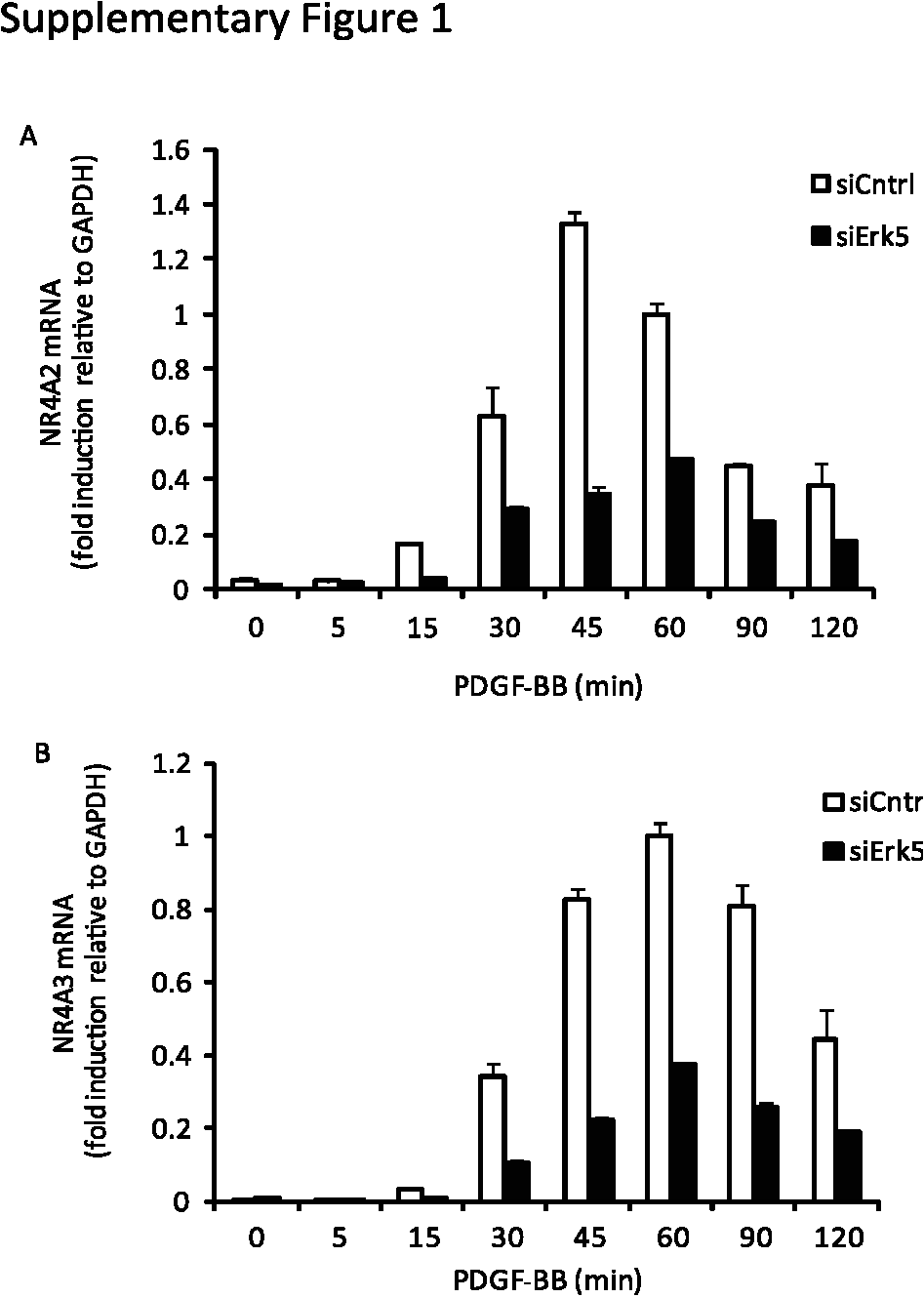

Supplement: Figure S1 — NR4A2 (Nurr1) and NR4A3 (NOR-1) mRNA are induced by PDGF-BB. NIH3T3 cells were treated with Erk5 siRNA or control siRNA and then stimulated by PDGF-BB (20 ng/ml) for indicated time periods. NR4A2 (A) and NR4A3 (B) mRNA levels were measured by quantitative RT-PCR. (TIF) [file pone.0109047.s001.tif]
